# Supplementary material for: Inflammation Adjustment by Two Methods Decreases the Estimated Prevalence of Zinc Deficiency in Malawi
Source: Nutrients. 2020 May 27;12(6):1563. doi: 10.3390/nu12061563 (PMC7352807; doi:10.3390/nu12061563)
Supplement: Supplementary file 1 [file nutrients-12-01563-s001.pdf]

nutrients

MDPI

Article

Inflammation Adjustment by Two Methods Decreases the Estimated Prevalence of Zinc Deficiency in Malawi

Blessings H. Likoswe [1,*], Felix P. Phiri [2,3], Martin R. Broadley [2], Edward J. M. Joy [4], Noel Patson [1,5], Kenneth M. Maleta [1] and John C. Phuka [1,*]

1 Department of Public Health, School of Public Health and Family Medicine, College of Medicine, University of Malawi, Private Bag 360, Chichiri, Blantyre 3, Malawi; ophumisa@gmail.com (N.P.); kmaleta@medcol.mw (K.M.M.)
2 School of Biosciences, University of Nottingham, Sutton Bonington Campus, Loughborough, Leicestershire LE12 5RD, UK; felixphiri8@gmail.com (F.P.P.); martin.broadley@nottingham.ac.uk (M.R.B.)
3 Department of Nutrition, HIV, and AIDS, Ministry of Health, Lilongwe 3, Malawi
4 Faculty of Epidemiology and Population Health, London School of Hygiene & Tropical Medicine, Keppel Street, London WC1E 7HT, UK; edward.joy@lshtm.ac.uk
5 School of Public Health, University of the Witwatersrand, Johannesburg 2193, South Africa
* Correspondence: blikoswe@medcol.mw (B.H.L.); jphuka@medcol.mw (J.C.P.); Tel.: +265-881-131-422/993-226-040 (J.C.P.)

Received: 16 April 2020; Accepted: 21 May 2020; Published: 27 May 2020

check for updates

Abstract: Serum zinc concentration (SZC) is used widely to assess population-level zinc status. Its concentration decreases during inflammatory responses, which can affect the interpretation of the results. This study aimed to re-estimate the prevalence of zinc deficiency in Malawi based on the 2015–2016 Malawi Micronutrient Survey (MNS) data, by adjusting SZC measures with markers of inflammation. SZC and inflammation data from 2760 participants were analysed. Adjustments were made using: (1) The Internal Correction Factor (ICF) method which used geometric means, and (2) The Biomarkers Reflecting Inflammation and Nutritional Determinants of Anemia (BRINDA) method, which used linear regression. Mean SZC values increased significantly when adjustments were made by either ICF or BRINDA ($p < 0.001$). The national prevalence of zinc deficiency decreased from 62% to 59%, after ICF adjustment, and to 52% after BRINDA adjustment. ICF and BRINDA values of SZC were highly correlated ($p < 0.001$, r = 0.99), but a Bland–Altman plot showed a lack of agreement between the two methods (bias of 2.07 µg/dL). There was no association between the adjusted SZC and stunting, which is a proxy indicator for zinc deficiency. Inflammation adjustment of SZC, using ICF or BRINDA, produces lower estimates of zinc deficiency prevalence, but the lack of agreement between the adjustment methods warrants further research. Furthermore, the lack of association between SZC and stunting highlights the need to explore other biomarkers and proxies of population zinc assessment. This study demonstrates the importance of considering inflammatory confounders when reporting SZC, to ensure accuracy and to support policy decision making.

Keywords: zinc; biomarkers; inflammation; C-reactive protein (CRP); alpha 1-acid glycoprotein (AGP); children; women of reproductive age

1. Introduction

Zinc is an important micronutrient in the human body and it plays various biochemical roles characterized by catalytic, structural and regulatory functions [1]. Zinc deficiency can cause ailments in all age groups such as poor growth, compromised immune system, cognitive impairment, delayed

sexual maturation and poor pregnancy outcomes [2]. Zinc deficiency is a global challenge, and more than 20% of children and women are estimated to be zinc deficient [3]. Although many countries lack national-level data on population zinc status, some countries such as Malawi conduct a periodic National Micronutrient Survey (MNS) in which biomarkers of zinc status are analysed.

Three main indicators (plasma/serum zinc concentration, dietary data and stunting) are recommended for use in the assessment of population-level zinc status [1]. Serum zinc is a biochemical indicator and it reflects individual zinc status to estimate zinc deficiency, compared to the other two indicators, which only predict the risk [2,4,5]. Despite this recommendation, serum zinc concentration (SZC) has limitations as a biomarker for zinc assessment, because it is affected by many factors such as inflammation, circadian variation and fasting status [2,6,7]. It is, nevertheless, the most commonly used indicator for population zinc status assessment; it was recently used in the Malawi MNS to assess zinc status of a nationally representative sample of men, women of reproductive age (WRA), school-age children (SAC), and pre-school children (PSC) [8]. Results from this survey indicated a zinc deficiency prevalence ranging from 60%–66% across these demographic groups. This prevalence is the highest recorded national prevalence in sub-Saharan Africa, and one of the highest globally [3]. However, the results were reported without adjusting for inflammation, which is an important confounder.

Inflammation induces a series of biochemical responses, which causes changes in serum and plasma concentration of acute phase reactants (APRs) in the body [9]. These APRs include nutrients such as zinc, which decrease in concentration, and ferritin, which increase in concentration. The SZC of an inflamed individual will thus be depressed below the actual value causing an artificial increase of the deficiency prevalence estimate. Several methods can be used to control for the effects of inflammation when reporting results of APR nutrients. The two most widely recommended methods use mathematical calculations to adjust the micronutrient concentrations [10–18]. These methods are reliant on an individuals' inflammation status, with inflammatory markers being measured at the same time as the micronutrient. The commonly measured biomarkers of inflammation in surveys are C-reactive protein (CRP) and alpha 1-acid glycoprotein (AGP), both of which are used to make the adjustments. The first recommended method is the Internal Correction Factor (ICF), which was suggested by Thurnham et al. [11] For this approach, geometric means are calculated and used to develop correction factors to adjust the micronutrient values. The other recommended method uses linear regression to adjust the values on a continuous scale. It was developed by the Biomarkers Reflecting Inflammation and Nutritional Determinants of Anemia (BRINDA) project, and is referred to as the BRINDA method [19]. Studies have shown that both methods can adjust concentrations of different micronutrients [11,13,18,20–25] and more specifically, concentrations of zinc in PSC [14,15], adults [16], and the elderly [12]. Currently, no studies have applied these methods, to adjust for zinc deficiency in a nationally representative sample of all age and gender groups.

The aim of this study was to re-estimate the prevalence of zinc deficiency in Malawi, based on the 2015–2016 Malawi MNS data, by adjusting SZC measures with markers of inflammation. The specific primary objectives were to: (a) re-estimate the prevalence of zinc deficiency in the MNS sample population by adjusting SZC values for inflammation, using ICF and BRINDA methods; and (b) compare the performance of ICF and BRINDA methods when used to adjust the MNS SZC values. A secondary objective was to assess the relationship between inflammation adjusted SZC values and stunting. It was expected that the prevalence of zinc deficiency in the Malawi MNS sample would decrease after adjusting for inflammation, using either method, and that ICF would decrease the values more modestly compared to BRINDA. Furthermore, a positive association between the adjusted values of SZC and stunting was expected, because stunting is also a recommended indicator of zinc assessment.

2. Material and Methods

Cross-sectional survey data from the Malawi MNS were used in this study. The Malawi MNS represents a sub-sample of the wider Malawi Demographic and Health Survey (MDHS), that ran in

2015–2016. The sampling design is presented in detail elsewhere [8]. In brief, a two-stage cluster design was used to sample participants from all 28 districts of the country. A cluster was a standard enumeration area, as defined by the Malawi population and housing census conducted in 2008. For each of the 105 selected clusters, 22 households in rural, and 20 households in urban areas, were randomly selected for inclusion in the study. In each household, men (ages 20–55 years), WRA (ages 15–49 years), SAC (ages 5–14 years) and PSC (ages 6–59 months) were recruited. Their serum samples were analysed at the Children's Hospital Oakland Research Institute (CHORI) in Oakland, USA, for zinc concentration, and the VitMin laboratory in Willstaet, Germany for CRP and AGP.

Datasets for the four age groups were accessed from the DHS program database. The calculated sample size for the MNS was 3243, but with a 90% response rate, data were collected on 3099 participants. An amended dataset was created to exclude participants that did not consent to venepuncture blood collection or were missing SZC, CRP or AGP values. Pregnant women and participants with negative values or values equal to 0 in the variables mentioned above were also removed. A SZC of 125 µg/dL was considered as the threshold, above which contamination was suspected; values above this level were also removed [26].

2.1. Adjustments of SZC

Normality of SZC data in the different age groups was assessed using measures of central tendency and histograms. The SZC values were adjusted separately for the four age groups using ICF and BRINDA methods. For ICF, a stepwise approach was used. Firstly, serum CRP and AGP concentrations were split into four categories: normal (CRP ≤ 5 mg/L and AGP ≤ 1.0 g/L), incubation (CRP > 5 mg/L and AGP ≤ 1.0 g/L), early convalescence (CRP > 5 mg/L and AGP > 1.0 g/L), and late convalescence (CRP ≤ 5 mg/L and AGP > 1.0 g/L). Then, the geometric means of each category were calculated, and geometric mean ratios were determined, using the formula presented below:

$$ICF_i = \frac{GM_{ref}}{GM_i} \tag{1}$$

where GM_{ref} is the reference/normal group of inflammation, and (i) denotes each of the three inflammation stages. Finally, ICFs were multiplied by each SZC observation in the respective category to calculate the adjusted SZC.

The BRINDA approach used multivariate regression models of $\log_e$-transformed SZC values (response variable), and $\log_e$-transformed CRP and AGP values (explanatory variables). Both CRP and AGP were included in each model. CRP and AGP internal reference (IR) values were calculated instead of using external references recommended by BRINDA. This was done to achieve uniformity since BRINDA only has IR values for WRA and PSC. The IR values are the $\log_e$-transformed maximum values of the lowest deciles of each inflammatory marker. Only participants with both CRP and AGP ($\log_e$-transformed) above the IR were adjusted using the equation presented below, whereas those with inflammatory markers below the IR were considered normal and not adjusted:

$$Adjusted\ serum\ zinc = \exp\left\{\ln\left(SZC_{unadj}\right) - \beta_1\left[\ln(CRP_{obs}) - \ln\left(CRP_{ref}\right)\right] - \beta_2\left[\ln(AGP_{obs}) - \ln\left(AGP_{ref}\right)\right]\right\} \tag{2}$$

where β_1 and β_2 are the coefficients derived from the regression equation. CRP_{obs} and AGP_{obs} denote the observed CRP and AGP values for the respective individual, and CRP_{ref} and AGP_{ref} denote the reference values. Regression results were interpreted as a percentage change in the response variable, after the percentage change in each explanatory variable. This followed the log-log interpretation of linear regression presented below:

$$\left(1.01^{\beta} - 1\right) * 100 \tag{3}$$

where β is the coefficient of the explanatory variable. Zinc deficiency was determined using the National Health and Nutrition Examination Survey (NHANES) SZC cut-offs, as shown in Table 1 below [27]. BRINDA values were back-transformed before applying these cut-offs.

Table 1. Recommended lower cut-offs (2.5th percentile) for serum zinc status, adapted from the National Health And Nutrition Examination Survey (NHANES) II data [27].

Serum Zinc Concentration Values µmol/L (µg/dL) [a]				
**Age Group**	**< 10 years**	**> 10 years**		
	**Children**	**Females**		**Males**
		**Non-Pregnant**	**Pregnant** [b]	
Morning Fasting	N/A	10.7 (70)	8.6 (56) 1st trimester:7.6 (50) 2nd/3rd trimester:	11.3 (74)
Morning non-fasting	9.9 (65)	10.1 (66)		10.7 (70)
Afternoon/evening	8.7 (57)	9.0 (59)		9.3 (61)

[a] Conversion factor: µmol/L = µg/dL ÷ 6.54. [b] Lower cut-offs controlled for time of day and fasting status.

2.2. Statistical Analyses

Analyses were conducted on age-distributed data and pooled data. Measures of central tendency and histograms were used to assess the normality of distribution. Point estimates were made using household-level sampling weights derived by MDHS. The MNS sample size was calculated to produce estimates on the national level, regional level (North, Central, and South) and type of domicile (Rural or Urban).

The means of the ICF and BRINDA adjusted SZC values were compared with the unadjusted values using ANOVA. The Bland–Altman method was used to compare performance of the two adjustment methods [28]. The clinical criteria for defining lack of agreement (bias) between the methods was a difference in SZC of 1 µg/dL. This value was derived from the maximum standard error of the cut-off values, which are used to define zinc deficiency in this study [27].

A sensitivity analysis was done to estimate the effect of normality of differences, on the limits of agreement. A robust regression approach designed to deal with non-uniform differences, suggested by Bland and Altman, was used [29]. The approach combines two regression models, to generate upper and lower limits of agreement (LOA). The first model plots the difference between methods (D) against the means of the two methods (M), to give:

$$D = x_0 + x_1 M \tag{4}$$

where x_0 is the intercept of the regression and x_1 is the coefficient. The obtained residuals (R) from this first model are then regressed against the means of the two methods (M) to produce the second linear equation, which is:

$$R = b_0 + b_1 M \tag{5}$$

In this model, b_0 and b_1 also denote the intercept and coefficient. If there is no significant relationship between the residuals and M, only the first model is used to calculate the limits of agreement. The limits of agreement can be calculated as:

$$D \pm 1.96 * SD \tag{6}$$

For model 1 only, where SD is the standard deviation of the residuals from the regression, and

$$D \pm 2.46R \tag{7}$$

for both models [29].

Stunting was determined in children under the age of 5 and defined as the length or height for age, which was less than −2.0 Z-score below the median value of the reference population [30]. Associations between stunting and adjusted SZC values were assessed using Pearson's Chi-square test

for independence, with Rao and Scott adjustments for complex survey design. A p-value of < 0.05 was considered significant for all the analyses.

2.3. Ethical Approval

Ethical clearance for the MNS was obtained from the National Health Science Research Committee in Malawi, reference number NHSRC 15/5/1436. For this secondary data analysis, ethical approval was obtained from the College of Medicine Research Ethics Committee (COMREC), protocol number P.01/20/2915.

3. Results

Of the 3099 participants surveyed in the MNS, 267 (8.7%) of participants did not consent to venepuncture blood collection during the survey, or were missing SZC, CRP or AGP values. Others were pregnant (n = 34; 1.1%), had negative SZC values (n = 2; < 1%), had SZC values equal to 0 (n = 1; < 1%), or were above the threshold, suggesting contamination (n = 35; 1.1%). These were removed and 2760 participants remained. Measures of central tendency indicated a normal distribution of SZC values for all age groups. Furthermore, both adjustment methods normalize distribution of the data, by working with $\log_e$-transformed variables.

Among the participants, PSC had the lowest SZC mean ± standard deviation (SD) of 58.4 ± 14.9 µg/dL before inflammation adjustments, as shown in Table 2. The highest mean concentration of 62.4 ± 15.8 µg/dL was recorded in SAC; however, it was not much higher than the mean concentration of men. Means of inflammatory markers were greatest in PSC, and lowest in WRA. The mean concentration of CRP in PSC was 6.3 ± 14.5 mg/L, and that of AGP was 1.4 ± 0.8 g/L. SAC also had a higher average concentration of CRP (4.3 ± 11.6 mg/L), however, they were below the threshold of inflammation. Similar results were observed in men and WRA, whose mean concentrations of CRP and AGP were below the threshold of inflammation.

Table 2. Summary of concentrations of SZC, CRP and AGP of the participants in the Malawi MNS.

Group	Mean SZC ± SD (µg/dL)	Mean CRP ± SD (mg/L)	Mean AGP ± SD (g/L)
Men	62.3 ± 14.1	2.7 ± 6.1	0.7 ± 0.4
WRA	60.1 ± 14.4	2.5 ± 8.5	0.7 ± 0.4
SAC	62.4 ± 15.8	4.3 ± 11.6	1.0 ± 0.6
PSC	58.4 ± 14.9	6.3 ± 14.5	1.4 ± 0.8
**Total**	**58.8 ± 15.0**	**5.9 ± 13.9**	**1.3 ± 0.8**

WRA: women of reproductive age, SAC: school-age children, PSC: pre-school children, CRP: C-reactive Protein, AGP: α-1-acid glycoprotein, SZC: serum zinc concentration.

3.1. Adjustment of Serum Zinc Concentration (SZC)

Table 3 presents the correction factors generated by the ICF approach. The correction factors increased SZC of all inflammation categories of men, SAC and PSC (correction factors were above 1, indicating a positive adjustment). The correction factor for WRA in the incubation stage was below 1. This resulted in a negative adjustment of the SZC in this stage. This category of WRA also had the highest mean SZC value of 62.7 ± 15.9 µg/dL compared to the other inflammation categories of the same age group. The overall mean SZC value for WRA was 60.1 ± 14.4 µg/dL, similar to the normal category (60.6 ± 15.2 µg/dL), and greater than the late convalescence category (56.1 ± 11.8 µg/dL). Back-transformed IR values used as cut-offs for BRINDA adjustment are also presented in Table 3. For AGP, the threshold for BRINDA adjustment was higher for children than adults. Among the children, the highest reference value for AGP (0.56) was obtained in PSC. The highest reference value for CRP was also derived in PSC; WRA had the second-highest IR followed by men, then SAC. All the IR values for AGP and CRP were below the cut-off of determining inflammation used by the ICF approach.

Table 3. Internal correction values for ICF and BRINDA that were used to adjust SZC values for inflammation.

Group	Inflammation Category	Correction Factors (ICF)	Internal Reference Values (BRINDA)	
			CRP	AGP
**Men**			0.13	0.37
	Normal	-		
	Incubation	1.060		
	Early	1.049		
	Late	1.058		
**WRA**			0.15	0.39
	Normal	-		
	Incubation	0.966		
	Early	1.008		
	Late	1.076		
**SAC**			0.12	0.46
	Normal	-		
	Incubation	1.070		
	Early	1.164		
	Late	1.037		
**PSC**			0.19	0.56
	Normal	-		
	Incubation	1.069		
	Early	1.105		
	Late	1.004		

Table 4 presents data from the $\log_e$-transformed models that were fit to adjust the SZC values using the BRINDA method. Four regression models were fitted for men, WRA, SAC and PSC. All linear models were plotted with SZC as the dependent factor and inflammatory markers as the predictor variables, and they indicated significant relationships. Model outputs showed that CRP was negatively associated with SZC in all age groups, whereas AGP was negatively associated with SZC in three age groups only. The multivariate models for SAC and PSC were highly significant ($p < 0.001$) compared to those of WRA and men ($p = 0.04$, $p = 0.01$). Only CRP was significantly associated with SZC when both CRP and AGP were included in the models for men, SAC, and PSC ($p < 0.001$). For WRA, both inflammatory markers were not significant in the multivariate model. For men, a 1% increase in AGP was associated with a 2% increase in SZC, and a 1% increase in CRP concentration was associated with a 3% decrease in SZC. Across demographic groups, CRP had the highest percentage association with SZC in men and the lowest percentage was observed in WRA. For WRA, a 1% increase of CRP and AGP were associated with an SZC decrease of 0.7% and 3%, respectively. However, the significance of this association was not very strong ($p = 0.040$). A 1% increase in AGP concentration of SAC was associated with a 4% decrease of their SZC. This was the highest percentage of change observed for AGP, across the demographic groups. SZC values of PSC were least strongly associated with AGP, as an increase of 1% of AGP was associated with a 1% decrease in SZC.

Table 4. Regression outputs of SZC versus inflammatory markers for different demographic groups.

Group	Log β-Coefficient		Intercept (95% CI)	R^2	*p*-Value
	CRP (95% CI)	AGP (95% CI)			
Men	−0.033 (−0.056, −0.010)	0.022 (−0.061, 0.105)	61.83 (58.79, 65.01)	0.03	0.013
WRA	−0.007 (−0.020, 0.006)	−0.030 (−0.076, 0.015)	57.70 (56.23, 59.21)	0.01	0.047
SAC	−0.021 (−0.032, −0.009)	−0.037 (−0.081, 0.006)	59.40 (58.29, 60.53)	0.05	<0.001
PSC	−0.026 (−0.035, −0.015)	−0.013 (−0.046, 0.020)	58.12 (57.21, 59.04)	0.04	<0.001

WRA: women of reproductive age, SAC: school-age children, PSC: pre-school children, CRP: C-reactive Protein, AGP: α-1-acid glycoprotein.3.2. Description of Serum Zinc Concentration (SZC) Data.

The distribution of SZC data values, before and after correcting for inflammation are shown in Figure 1. The mean ± SD SZC was 58.8 ± 15.0 µg/dL before adjustment, and it increased to 60.2 ± 15.4 µg/dL after ICF adjustment, and 62.4 ± 15.8 µg/dL after BRINDA adjustment. The medians of the distribution were also close to the means, at 56.3 µg/dL, 60.6 µg/dL, and 60.5 µg/dL for unadjusted, ICF, and BRINDA adjusted, respectively. A greater magnitude of change was observed on the high concentration end of SZC compared to the low concentration end. The maximum observed SZC value (125.0 µg/dL), changed by approximately 15 µg/dL, whereas the lowest observed value (15 µg/dL) changed by approximately 1 µg/dL after both adjustment methods.

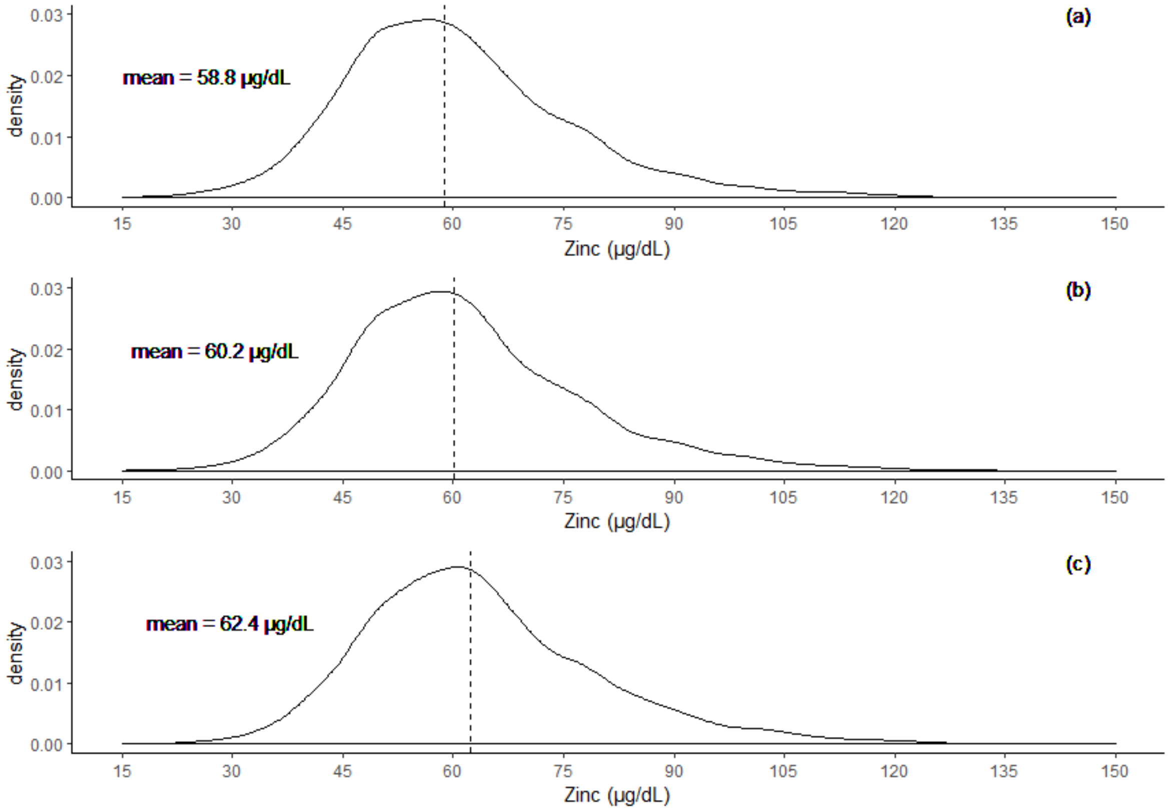

Figure 1. Density distribution of SZC values (**a**) before adjustment, (**b**) after adjusting with the ICF method, and (**c**) after adjusting with BRINDA method. Dashed lines correspond to mean values.

3.2. Prevalence Point Estimates

Prevalence estimates of SZC deficiency, before and after adjustment, are shown in Table 5. The unadjusted estimates presented here may vary from the figures presented in the MNS report, due to differences in sample size as a result of data cleaning. The national prevalence of zinc deficiency was 62% before adjustments and it decreased to 59% after ICF correction, and 52% after BRINDA correction. Before adjustment, men had the highest prevalence of zinc deficiency across all the age groups, followed by WRA. This order did not change following ICF adjustment, but it was reversed when the values were adjusted by BRINDA. SAC had the largest decrease in prevalence estimate when both ICF (6%) and BRINDA (11%) adjustments were made. Men had a modest decrease of 2% following ICF adjustment, but a larger decrease with BRINDA (10%). Decreases in the prevalence estimates of PSC followed a similar pattern as that of men. Overall, there were limited decreases in zinc deficiency estimates of WRA with both methods.

Table 5. Prevalence estimates of serum zinc deficiency and inflammation in Malawi presented across multiple age groups.

	Inflammation [a]	Serum Zinc [b]		
		Unadjusted	ICF	BRINDA
	Prev. (95% CI)	Prev. (95% CI)	Prev. (95% CI)	Prev. (95% CI)
Men	14 (9, 22)	71 (60, 79)	69 (60, 78)	61 (48, 72)
WRA	14 (10, 17)	66 (59, 72)	65 (58, 71)	63 (56, 70)
SAC	34 (29, 39)	56 (48, 63)	51 (44, 59)	45 (38, 52)
PSC	57 (51, 62)	61 (55, 67)	58 (52, 64)	52 (46, 58)
**Total**	**52 (47, 57)**	**62 (56, 67)**	**59 (53, 64)**	**52 (46, 58)**

[a] Inflammation cut-offs: CRP ≤ 5 mg/L and AGP ≤ 1 g/L [11], [b] zinc deficiency cut-offs presented in Table 1.

The prevalence of inflammation was determined based on the cut-offs suggested by Thurnham et al. [11], which combined CRP and AGP measures. PSC had the highest prevalence of inflammation (57%) among the demographic groups, followed by SAC (34%). However, the change in zinc deficiency of PSC was not reflective of this high inflammation rate of that age group. Men had a very low prevalence of inflammation (14%), but had the largest magnitude of change following BRINDA adjustments, compared to the other demographic groups. Data on zinc deficiency prevalence, stratified by region, type of domicile and gender, is presented in Table S1. These data show no statistical differences in zinc prevalence between the different regions or rural and urban dwellers ($p > 0.050$).

3.3. Differences in SZC Across The Three Groups

There was a significant difference in mean SZC of the unadjusted, ICF and BRINDA adjusted values ($p < 0.001$). Post-hoc results from the Tukey HSD test indicated that the mean SZC following ICF adjustment was higher than the mean of the unadjusted values ($p < 0.010$), with a mean difference of 1.21 µg/dL (95% CI: 0.22, 2.19). BRINDA adjusted values were also significantly higher than unadjusted values ($p < 0.001$), with a bigger difference of 3.24 µg/dL (95% CI: 2.25, 4.22) between the means of those two groups.

3.4. Assessment of Agreement Methods

There was a strong correlation between values adjusted by ICF and BRINDA methods, as shown in Figure 2, ($R^2 = 0.99$). There was thus a strong positive linear relationship between the values adjusted by ICF and those of BRINDA. The regression equation was $ICF = 0.19 + 0.96 \times BRINDA$, indicating that for each 1 µg/dL increase in BRINDA values, there was a 0.96 µg/dL increase in ICF values. Differences between the methods were plotted against the means of the paired values in the Bland–Altman plot. The data points showed a scattered but downward pattern, which grew wider as the SZC increased. The plot showed a bias of −2.07 µg/dL (95% CI: −2.15, −2.00), indicating a lack of agreement between the methods. On average, BRINDA adjusted SZC values were greater than ICF adjusted values by more than 2 µg/dL, which is more informative than the linear equation. This value exceeds the limit of clinical significance of 1 µg/dL, defined a priori. Furthermore, the confidence interval, as presented, did not include the line of equality (0), which confirmed the significance of the systematic difference between ICF and BRINDA approaches. The upper LOA was 1.82 µg/dL (95% CI: 1.69, 1.95), and the lower LOA was −5.96 µg/dL (95% CI: −6.09, −5.84), indicating that for 95% of the data points, ICF-adjusted SZC values were between 1.82 µg/dL greater or 5.96 µg/dL less than BRINDA-adjusted SZC values. There was an overlap of the 95% confidence intervals of the estimates from both methods, suggesting that the true SZC values may lie between the estimates from the two methods.

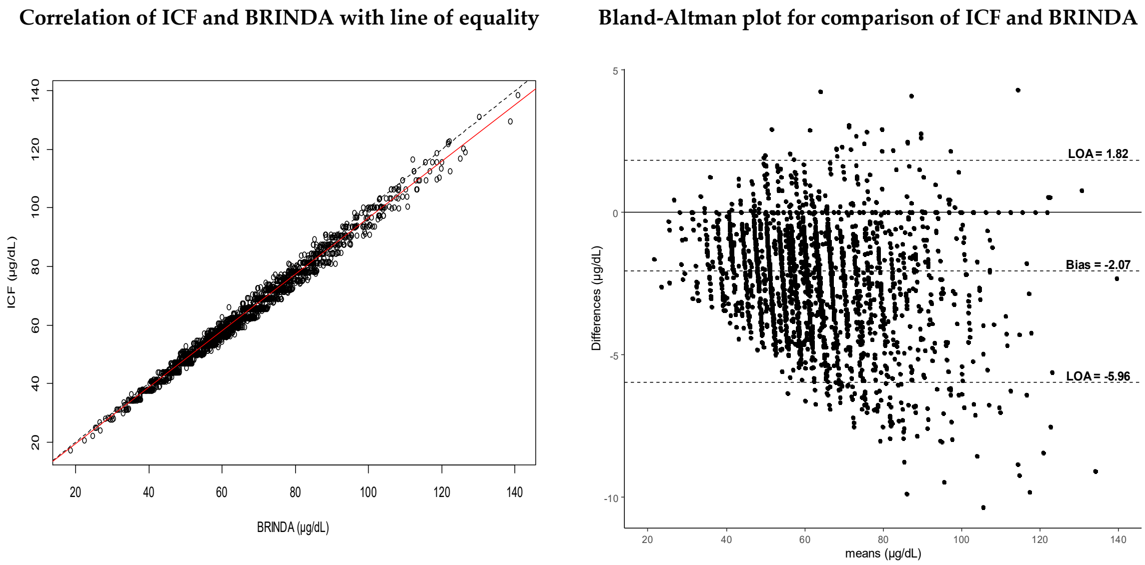

Figure 2. Method comparisons between ICF and BRINDA approaches, presented as a linear correlation in the left panel and Bland–Altman plot in the right panel.

The sensitivity analysis derived an intercept of $x_0 = -0.26$ and a coefficient of $x_1 = -0.03$, which were used to calculate the regression LOAs. At the highest point of the regression line, upper LOA was 3.01 [2.89, 3.14] µg/dL and lower LOA was −4.57 [−4.67, −4.44] µg/dL. At the lowest point of the regression line, upper LOA was −0.49 [−0.62, −0.37] µg/dL and the lower LOA was −8.07 [−8.20, −7.95] µg/dL. This gave a difference of 7.58 µg/dL between the upper and lower LOAs, which was similar to the difference between the original LOAs (7.78 µg/dL). The difference between the parametric and non-parametric LOAs was 0.2 µg/dL, which is not clinically meaningful; the regression graph is presented as a supplement in Figure S1.

3.5. Association Relationships between Serum Zinc Concentration (SZC) and Stunting

There was no significant association between SZC and stunting in PSC. This was true for both ICF adjusted SZC ($X^2 = 0.09$, $p = 0.828$) and BRINDA adjusted SZC ($X^2 = 0.05$, $p = 0.864$). Stunting was assessed in 1050 PSC, of whom 34% were stunted. When stratified by gender, the prevalence was 35% and 33% in female and male children, respectively. PSC aged 6–23 months (n = 314) had a lower stunting prevalence of 24%, compared to those aged 24–59 months (n = 736), who had a prevalence of 39%. Fifty-nine percent of all the PSCs were zinc deficient after adjusting for ICF, and 34% of them were also stunted. Furthermore, 35% PSC, with normal SZC in this adjustment method, were also stunted. These results showed no difference in the prevalence of stunting between zinc deficient children, and those with normal SZC. For BRINDA, 52% of PSC were zinc deficient and 34% of these were stunted, whereas 34% of PSC with normal SZC were also stunted. There was also no difference in the prevalence of stunting between PSC with normal zinc levels, and those who were deficient after adjusting with BRINDA.

4. Discussion

The prevalence of zinc deficiency in Malawi was re-estimated by correcting SZCs for inflammation, using existing methods among a nationally representative sample of the Malawi population. Both methods reduced the prevalence of zinc deficiency in the Malawi population from 62% to 59% with the ICF method, and from 62% to 52% with the BRINDA method. These results are consistent with current understanding that SZCs are depressed during inflammation [31]. Despite the reduction, zinc deficiency was still widespread. Siyame et al. previously reported a high zinc deficiency prevalence (>90%), among women from two rural locations in Malawi [32]. Huddle et al. found a

zinc deficiency prevalence of > 40% among pregnant women, also living in a rural Malawi setting [33]. The estimated prevalence of deficiency in this current study varied between demographic groups. At 52%, the prevalence of zinc deficiency in PSC in Malawi was the second-highest across African countries [3]. SAC had a lower prevalence estimate (45%) compared to other demographic groups, across both methods of adjustment. This is still above the WHO threshold of 20%, above which a public health intervention aimed to improve zinc status is recommended [34]. The reduction in national level zinc deficiency prevalence after ICF was only 3%, but for BRINDA, it was much higher (10%). A 10% decrease in prevalence of zinc deficiency is significant for public health stakeholders, because it can affect resource allocation for intervention projects. Furthermore, if the prevalence of zinc deficiency in this population were closer to the threshold of public health concern (20%), a 10% change could influence the decision of whether a public health intervention is necessary or not. Inflammation adjustment of SZC is thus necessary to avoid overestimation of the problem.

The 2015–2016 MNS was the first national-level assessment of zinc deficiency in Malawi using biochemical indicators. Previous studies have used dietary zinc supply data to estimate the risk of zinc deficiency. These risks were reported as 33% in 2009 [35], and 57% in 2010–2011 [36], based on nationally representative data from other surveys. The latter estimate was similar to what is observed in the present study. Inadequate dietary zinc supplies are likely to be widespread in Malawi, since diets are primarily plant-based and with high phytate content, which reduces zinc bio-availability [37]. A phytate: zinc ratio of > 15 is associated with low absorption of zinc [2]. Individual studies have shown that diets in Malawi have high phytate: zinc molar ratios, e.g., ranging from 17–24 [32] to 30.2 [36]. These findings are not surprising, given that the Malawian diet is monotonous, with most of the calorie contributions coming from maize, which has a phytate: zinc molar ratio of 23 [37]. Soil type is also an important factor to consider, because it affects micronutrient concentrations in the food and consequently the dietary intake levels [38]. For example, Joy et al. found that zinc concentration of some food items sampled in Malawi were affected by the type of soil where it was grown [39]. This highlights the necessity of further investigating the zinc deficiencies with respect to spatial variation.

Non-dietary factors, such as environmental enteric dysfunction [40], acute and persistent diarrhea [41,42], and intestinal helminthic infections [43], can affect the absorption of zinc, and consequently SZC. These illnesses have been detected in some Malawian population groups [40]. Zinc deficiency has further been linked to genetic disorders such as sickle cell disease and thalassemia [44], but also other micronutrients such as iron and calcium, all of which are endemic in tropical countries, and may have contributed to the high levels if zinc deficiency [1]. The prevalence of inflammation was high, especially in PSC and SAC. Inflammation usually occurs in response to an infection, though other cases may be subclinical. In this study sample, it may have been caused by malaria, which was reported in 28% of PSC and 42% of SAC or urinary schistosomiasis, which was found in 30% of PSC and 22% of SAC. Furthermore, anemia and iron deficiency are also strongly associated with infection [45], and both were prevalent in the study population. This is all consistent with the observation that zinc deficiency and inflammation prevalence were high in the MNS sample population.

The SZC values increased more when adjusted by the BRINDA method compared to the ICF method. Similar results have been reported elsewhere [12,13]. Different methodological approaches may have contributed to this poor agreement. Firstly, ICF uses combined thresholds of CRP (≤ or >5 mg/L) and AGP (≤ or >1 g/L), to identify inflamed individuals for adjustment [11]. On the other hand, BRINDA uses IR values presented in Table 3 as the cut-off points for adjustment. These values created a lower threshold than that used by ICF, resulting in more serum values (about 80%) being corrected for inflammation. The BRINDA group highlights a linear relationship between the inflammatory markers and APR nutrients [21–23], which presents altered APR nutrient concentrations at levels below the internationally accepted inflammation cut-offs of CRP (≤5 mg/L) and AGP (≤1.0 g/L) [46]. This lack of consensus for cut-off marks for inflammation, needs to be explored further, to improve agreement of the adjustment methods.

The second methodological difference is the categorical adjustment by ICF, as opposed to the continuous adjustment by BRINDA. Reports in the literature suggest that the extent of SZC depression during inflammation is based on stage of inflammation but also severity of infection, which is characterized by concentration of the inflammatory markers [16,47–49]. Based on this, an individual who is in incubation (i.e., CRP > 5 mg/L and AGP ≤ 1.0 g/L), with a CRP concentration of 6 mg/L, would be expected to have a lower magnitude of zinc depression compared to one with a CRP of 12 mg/L. As such, adjusting them using the same correction factor may under-adjust the more severe case. Similarly, if the adjustment approach does not take inflammatory stage into account, it may result in other cases being over or under-adjusted. In the context of these two methods, neither is likely to adjust SZCs properly, since the ICF method focuses on stages of inflammation and disregards severity, whilst the BRINDA method, through the linear adjustment, focuses on severity and disregards inflammation stage. Both approaches can thus be improved if they incorporate the severity of the inflammatory response in the different inflammation stages. One way of approaching this would be to adjust the values using linear regression, but separately for each inflammatory stage. This could improve the accuracy of each method, and consequently their agreement. Furthermore, there is a need to explore and incorporate other markers of inflammation that better determine the severity of the case. This would simplify the integration of severity in the inflammation stages.

For ICF adjustments, the correction factors that were derived using ICF did not differ much from those found in previous studies, which adjusted SZC. For instance, Karakochuk et al. [14], found correction factors of 1.01, 1.15, and 1.07 in incubation, early, and late convalescence categories of PSC respectively, which is similar to what was observed and reported in Table 3. Mburu et al. [16] also applied the ICF method to adjust SZC values of adults, and their correction factors ranged between 1.08 and 1.27, which was slightly higher than what was observed in this current study, for both men and WRA. Notably, their study only included participants who had tested positive for HIV, which introduces some bias in terms of susceptibility to infections.

The incubation category for WRA had the highest mean of SZC values across this age group, resulting in a correction factor of < 1. Similar results have been observed in other studies that adjusted for inflammation in WRA and PSC. Macdonell et al., Grant et al. and Fiorentino et al. all found a correction factor of > 1 for soluble transferrin receptor, which is a positive APR [12,20,25]. Furthermore, a correction factor of < 1 was reported for retinol-binding protein, which is a negative APR [25]. The correction factor is based on the ratio of inflammation group against the normal group, and an inverse ratio will have an opposite effect on the results. The aim of adjusting negative APRs is to increase the concentration values that are reduced by inflammation. Therefore, the reduction of other values in the same data negates the purpose of this exercise. This poses the question of whether it is necessary to adjust categories that exhibit such inverse ratios, or if APR normalization by ICF should focus only on the categories which need the adjustment. These inverse ratios were mostly observed in the incubation category in all the studies referenced above, which shows a gap in the general understanding of the inflammatory response and APR interactions during early stages of inflammation. It may be possible that SZC does not drop as much during the first 48 h of inflammation, however, this has not been clearly described in the literature, and further research is needed.

Zinc plays an important role in growth, and poor zinc status in children may affect bone growth, leading to stunting. When the prevalence of stunting is > 20%, it is indicative of an elevated risk of zinc deficiency in the population [34]. The results show a stunting prevalence above this threshold in PSC, but no association between stunting (yes/no), and adjusted SZC values of both ICF ($X^2 = 0.09$, $p = 0.828$) and BRINDA ($X^2 = 0.05$, $p = 0.864$) methods. This has also been reported in other studies, such as Hess et al., who reviewed stunting and SZC data from national surveys of 20 countries and found conflicting results when comparing zinc deficiency with stunting prevalence estimates [3]. The comparison was made in 19 of the 20 surveys and graphical results showed that stunting under-presented the severity of zinc deficiency in some of the countries, despite blood analyses showing high deficiency rates. For countries like Nigeria and Afghanistan, stunting levels indicated a low risk of zinc deficiency (i.e.,

a stunting prevalence of < 20%), whereas SZC showed a zinc deficiency prevalence of > 40% in both countries. The risk of zinc deficiency was thus poorly predicted by stunting in most of these surveys. Individual studies have not been able to determine direct relationships between SZC and stunting using different approaches [14,50–52].

Causative factors of stunting have been inadequately explored in the literature, which poses a challenge for its use as a functional indicator of zinc deficiency [50,53]. Zinc, as a type II nutrient, contributes to growth through cell differentiation, and its deficiency induces growth restriction, because the body prioritizes homeostatic balances over growth [54]. There are about nine other nutrients of this type II, e.g., phosphorus, magnesium and amino acids which also contribute to tissue generation and growth. A balanced supply of all these nutrients is necessary to ensure growth as such. Although zinc deficiency causes growth faltering, it is not always solely responsible for poor growth. If there was an adequate supply of zinc, but low supply of the other type II nutrients in a population, stunting would still be observed. Whilst stunting is a potentially useful indicator of zinc deficiency, it is less sensitive in populations with multiple nutrient deficiencies. Furthermore, stunting is only useful for predicting zinc deficiency in children under the age of 5, whereas this study showed that zinc deficiency is common in all age groups.

The recommendation to combine SZC with dietary data cannot be easily achieved at a national level, because of various challenges. Comprehensive individual-level dietary data is not available at national level in Malawi. Food consumption data is collected in household surveys, but this is of limited value, given the interest in defining zinc deficiency among specific age or demographic groups. Furthermore, many studies have shown no correlation between dietary zinc intake and SZC [55], which also limits the accuracy of estimating zinc deficiency even when both indicators are used. These factors highlight the challenges of population zinc assessment and support the findings of Lowe et al. [5], that more research is required to identify novel zinc biomarkers.

Reports in the literature suggest that prior zinc deficiency could be the cause of increased inflammation as opposed to inflammation lowering SZC [47]. The present study uses data from a cross-sectional study design, which builds on the association of SZC and inflammation, making it impossible to determine causality between these two, and this is a limitation of the study. Nonetheless, the causal relationship between SZC and inflammation is worth investigating, through a controlled experiment in future research. It is proven that inflammation causes SZC to decrease, however, there is a high possibility that inflammation adjustment is only necessary for a fraction of the inflamed persons. Better understanding of the causal relationship would contribute greatly to improvement of the adjustment methods. Data quality issues were also identified in the study, as initial data exploration showed clumping of the SZC values across all age groups. This may have occurred due to various reasons such as data transfer from assay instruments, or reagent correction adjustments in the lab. This poses another limitation of this study and highlights some of the potential challenges that occur with the use of SZC alone as a biomarker for zinc deficiency.

5. Conclusions

Overall, adjusting for inflammation increased the SZC of the study participants, and thereby decreased the estimated prevalence of zinc deficiency in Malawi. The prevalence of zinc deficiency in the country remained high across all age groups, highlighting the need for nutrition policies and interventions to combat this problem. The biggest changes were observed after BRINDA adjustment, whereas ICF gave more modest changes. There were important differences in the results, depending on the adjustment method employed. Further research is needed to explore this difference, and to understand the mechanisms behind inflammation and SZC, which will help improve the inflammation adjustment methodologies. Stunting in PSC was not associated with SZC. While stunting prevalence may be useful to predict the risk of zinc deficiency, it may underestimate the severity of the problem, and there is a need to develop better indicators of zinc deficiency.

Supplementary Materials: The following are available online at http://www.mdpi.com/2072-6643/12/6/1563/s1, Table S1, Prevalence estimates of serum zinc deficiency and inflammation in Malawi, stratified by region, domicile type and gender, Figure S1, Bland-Altman regression plot for sensitivity analysis of differences against means of ICF and BRINDA.

Author Contributions: Conceptualization, B.H.L., E.J.M.J., J.C.P., K.M.M. and F.P.P.; Data acquisition, B.H.L. and F.P.P.; methodology, B.H.L., N.P., J.C.P. and K.M.M.; Formal analysis, B.H.L. and N.P.; funding acquisition, E.J.M.J. and M.R.B.; writing—original draft preparation, B.H.L., J.C.P. and K.M.M.; writing—review and editing, B.H.L., E.J.M.J., M.R.B., N.P., J.C.P. and K.M.M.; All authors have read and agreed to the published version of the manuscript.

Funding: This research was funded by the Bill & Melinda Gates Foundation, through the GeoNutrition project (INV-009129). The funders were not involved in any stages of the study design or implementation.

Acknowledgments: Special thanks should go to Vincent Samuel, Jessie Khaki, Professor Elizabeth Allen and Professor Suzanne Filteau, for their insights in the methodology and the statistical component of the study. Appreciation also goes to the Malawi National Statistics Office and the DHS program, for facilitating data access for this study.

Conflicts of Interest: Authors declare no conflict of interest.

References

1. King, J.C.; Brown, K.H.; Gibson, R.S.; Krebs, N.F.; Lowe, N.M.; Siekmann, J.H.; Raiten, D.J. Biomarkers of Nutrition for Development (BOND) - Zinc Review. *J. Nutr.* **2016**, *146*, 858S–885S. [CrossRef]
2. Brown, K.H.; Rivera, J.A.; Bhutta, Z.; Gibson, R.S.; King, J.C.; Ruel, M.T.; Brittmarie, S.; Wasanwisut, E.; Hotz, C. International Zinc Nutrition Consultative Group (IZiNCG) Technical Document #1. Assessment of the Risk of Zinc Deficiency in Populations and Options for Its Control. *Food Nutr. Bull.* **2004**, *25*, s91–s203.
3. Hess, S.Y. National risk of zinc deficiency as estimated by national surveys. *Food Nutr. Bull.* **2017**, *38*, 3–17. [CrossRef] [PubMed]
4. Walker, C.L.F.; Black, R.E. Functional indicators for assessing zinc deficiency. *Food Nutr. Bull.* **2007**, *28*, S454–S479. [CrossRef] [PubMed]
5. Lowe, N.M.; Fekete, K.; Desci, T. Methods of assessment of zinc status in humans: A systematic review. *Am. J. Clin. Nutr.* **2009**, *89*, 2040s–2051s. [CrossRef]
6. King, J.C. Zinc: An essential but elusive nutrient. *Am. J. Clin. Nutr.* **2011**, *94*, 679S. [CrossRef]
7. Wieringa, F.T.; Dijkhuizen, M.A.; Fiorentino, M.; Laillou, A.; Berger, J. Determination of zinc status in humans: Which indicator should we use? *Nutrients* **2015**, *7*, 3252–3263. [CrossRef]
8. National Statistical Office(NSO); Community Health Sciences Unit(CHSU); Centers for Disease Control and Prevention (CDC); Emory University. *Malawi Micronutrient Survey 2015-16*; NSO: Atlanta, GA, USA; CHSU: Atlanta, GA, USA; CDC: Atlanta, GA, USA; Emory University: Atlanta, GA, USA, 2017.
9. Galloway, P.; McMillan, D.C.; Sattar, N. Effect of the inflammatory response on trace element and vitamin status. *Ann. Clin. Biochem.* **2000**, *37*, 289–297. [CrossRef]
10. Namaste, S.M.; Aaron, G.J.; Varadhan, R.; Peerson, J.M.; Suchdev, P.S. Methodologic approach for the Biomarkers Reflecting Inflammation and Nutritional Determinants of Anemia (BRINDA) project. *Am. J. Clin. Nutr.* **2017**, *106*, 333S–347S.
11. Thurnham, D.I.; Mccabe, L.D.; Haldar, S.; Wieringa, F.T.; Northrop-clewes, C.A.; Mccabe, G.P. Adjusting plasma ferritin concentrations to remove the effects of subclinical inflammation in the assessment of iron deficiency: A meta-analysis. *Am. J. Clin. Nutr.* **2010**, *92*, 546–555. [CrossRef]
12. MacDonell, S.O.; Miller, J.C.; Harper, M.J.; Reid, M.R.; Haszard, J.J.; Gibson, R.S.; Houghton, L.A. A comparison of methods for adjusting biomarkers of iron, zinc, and selenium status for the effect of inflammation in an older population: A case for interleukin 6. *Am. J. Clin. Nutr.* **2018**, *107*, 932–940. [CrossRef] [PubMed]
13. Mei, Z.; Namaste, S.M.; Serdula, M.; Suchdev, P.S.; Rohner, F.; Flores-Ayala, R.; Addo, O.Y.; Raiten, D.J. Adjusting total body iron for inflammation: Biomarkers Reflecting Inflammation and Nutritional Determinants of Anemia (BRINDA) project. *Am. J. Clin. Nutr.* **2017**, *106*, 383S–389S. [PubMed]

14. Karakochuk, C.D.; Barr, S.I.; Boy, E.; Bahizire, E.; Tugirimana, P.L.; Akilimali, P.Z.; Houghton, L.A.; Green, T.J. The effect of inflammation on serum zinc concentrations and the prevalence estimates of population-level zinc status among Congolese children aged 6–59 months. *Eur. J. Clin. Nutr.* **2017**, *71*, 1467–1470. [CrossRef] [PubMed]
15. Diana, A.; Haszard, J.J.; Purnamasari, D.M.; Nurulazmi, I.; Luftimas, D.E.; Rahmania, S.; Nugraha, G.I.; Erhardt, J.; Gibson, R.S.; Houghton, L.; et al. Iron, zinc, vitamin A and selenium status in a cohort of Indonesian infants after adjusting for inflammation using several different approaches. *Br. J. Nutr.* **2017**, *118*, 830–839. [CrossRef]
16. Mburu, A.S.W.W.; Thurnham, D.I.; Mwaniki, D.L.; Muniu, E.M.; Alumasa, F.M. The influence of inflammation on plasma zinc concentration in apparently healthy, HIV Kenyan adults and zinc responses after a multi-micronutrient supplement. *Eur. J. Clin. Nutr.* **2010**, *64*, 510–517. [CrossRef]
17. Lim, K.; Booth, A.; Szymlek-Gay, E.A.; Gibson, R.S.; Bailey, K.B.; Irving, D.; Nowson, C.; Riddell, L. Associations between dietary iron and zinc intakes, and between biochemical iron and zinc status in women. *Nutrients* **2015**, *7*, 2983–2999. [CrossRef]
18. Mwangi, M.N.; Echoka, E.; Knijff, M.; Kaduka, L.; Werema, B.G.; Kinya, F.M.; Mutisya, R.; Muniu, E.M.; Demir, A.Y.; Verhoef, H.; et al. Iron status of Kenyan pregnant women after adjusting for inflammation using BRINDA regression analysis and other correction methods. *Nutrients* **2019**, *11*, 420. [CrossRef]
19. Suchdev, P.S.; Namaste, S.M.L.; Aaron, G.J.; Raiten, D.J.; Brown, K.H.; Flores-ayala, R. Overview of the Biomarkers Reflecting Inflammation and Nutritional Determinants of Anemia (BRINDA) Project. *Adv. Nutr.* **2016**, *7*, 349–356. [CrossRef]
20. Grant, F.K.E.; Suchdev, P.S.; Flores-ayala, R.; Conrad, R.; Ramakrishnan, U.; Ruth, L.J.; Martorell, R.; Program, H.S. Correcting for inflammation changes estimates of iron deficiency among rural Kenyan pre-school children. *J. Nutr.* **2016**, *142*, 105–111. [CrossRef]
21. Larson, L.M.; Namaste, S.M.; Williams, A.M.; Engle-Stone, R.; Addo, O.Y.; Suchdev, P.S.; Wirth, J.P.; Temple, V.; Serdula, M.; Northrop-Clewes, C.A. Adjusting retinol-binding protein concentrations for inflammation: Biomarkers Reflecting Inflammation and Nutritional Determinants of Anemia (BRINDA) project. *Am. J. Clin. Nutr.* **2017**, *106*, 390S–401S.
22. Rohner, F.; Namaste, S.M.; Larson, L.M.; Addo, O.Y.; Mei, Z.; Suchdev, P.S.; Williams, A.M.; Sakr Ashour, F.A.; Rawat, R.; Raiten, D.J.; et al. Adjusting soluble transferrin receptor concentrations for inflammation: Biomarkers Reflecting Inflammation and Nutritional Determinants of Anemia (BRINDA) project. *Am. J. Clin. Nutr.* **2017**, *106*, 372S–382S.
23. Namaste, S.M.; Rohner, F.; Huang, J.; Bhushan, N.L.; Flores-Ayala, R.; Kupka, R.; Mei, Z.; Rawat, R.; Williams, A.M.; Raiten, D.J.; et al. Adjusting ferritin concentrations for inflammation: Biomarkers Reflecting Inflammation and Nutritional Determinants of Anemia (BRINDA) project. *Am. J. Clin. Nutr.* **2017**, *106*, 359S–371S.
24. Cichon, B.; Ritz, C.; Fabiansen, C.; Christensen, V.B.; Filteau, S.; Friis, H.; Kaestel, P. Assessment of regression models for adjustment of iron status biomarkers for inflammation in children with moderate acute malnutrition in Burkina Faso. *J. Nutr.* **2016**, *147*, 125–132. [CrossRef]
25. Fiorentino, M.; Perignon, M.; Kuong, K.; Chamnan, C.; Berger, J.; Wieringa, F.T. Subclinical inflammation affects iron and Vitamin A but not zinc status assessment in Senegalese children and Cambodian children and women. *Public Health Nutr.* **2018**, *21*, 1266–1277. [CrossRef]
26. Bayse, D.D.; Gunter, E.W.; Neese, J.W.; Turner, W.E. *Laboratory Procedures used by the Clinical Division, Centers for Disease Control for the Second Health and Nutrition Examination Survey (NHANES II) 1976–1980*; U.S Department of Health and Human Services: Atlanta, GA, USA, 1985.
27. Hotz, C.; Peerson, J.M.; Brown, K.H. Suggested lower cutoffs of serum zinc concentrations for assessing zinc status: Reanalysis of the second National Health and Nutrition Examination Survey data (1976–1980). *Am. J. Clin. Nutr.* **2003**, *78*, 756–764. [CrossRef]
28. Bland, J.M.; Altman, D.G. Statistical methods for assessing agreement between two methods of clinical measurement. *Lancet* **1986**, *327*, 307–310. [CrossRef]
29. Bland, J.M.; Altman, D.G. Measuring agreement in method comparison studies. *Stat. Methods Med. Res.* **1999**, *8*, 135–160. [CrossRef]
30. World Health Organization. *Physical Status: The Use and Interpretation of Anthropometry*; World Health Organization: Geneva, Switzerland, 1995; Volume 1995.

31. Ghashut, R.A.; McMillan, D.C.; Kinsella, J.; Vasilaki, A.T.; Talwar, D.; Duncan, A. The effect of the systemic inflammatory response on plasma zinc and selenium adjusted for albumin. *Clin. Nutr.* **2016**, *35*, 381–387. [CrossRef]
32. Siyame, E.W.P.; Hurst, R.; Wawer, A.A.; Young, S.D.; Broadley, M.R.; Chilimba, A.D.C.; Ander, L.E.; Watts, M.J.; Chilima, B.; Gondwe, J.; et al. A high prevalence of zinc-but not iron-deficiency among women in Rural Malawi: A cross-sectional study. *Int. J. Vitam. Nutr. Res.* **2013**, *83*, 176–187. [CrossRef]
33. Huddle, J.-M.; Gibson, R.S.; Cullinan, T.R. Is zinc a limiting nutrient in the diets of rural pregnant Malawian women? *Br. J. Nutr.* **1998**, *79*, 257–265. [CrossRef]
34. de Benoist, B.; Darnton-Hill, I.; Davidsson, L.; Fontaine, O.; Hotz, C. Conclusions of the joint WHO/UNICEF/IAEA/IZiNCG interagency meeting on zinc status indicators. *Food Nutr. Bull.* **2007**, *28*, S480–S484. [CrossRef]
35. Joy, E.J.M.; Ander, E.L.; Young, S.D.; Black, C.R.; Watts, M.J.; Chilimba, A.D.C.; Chilima, B.; Siyame, E.W.P.; Kalimbira, A.A.; Hurst, R.; et al. Dietary mineral supplies in Africa. *Physiol. Plant.* **2014**, *151*, 208–229. [CrossRef]
36. Joy, E.J.M.; Kumssa, D.B.; Broadley, M.R.; Watts, M.J.; Young, S.D.; Chilimba, A.D.C.; Ander, E.L. Dietary mineral supplies in Malawi: Spatial and socioeconomic assessment. *BMC Nutr.* **2015**, *1*, 42. [CrossRef]
37. Manary, M.J.; Hotz, C.; Krebs, N.F.; Gibson, R.S.; Westcott, J.E.; Broadhead, R.L.; Hambidge, K.M. Zinc homeostasis in Malawian children consuming a high-phytate, maize-based diet. *Am. J. Clin. Nutr.* **2002**, *75*, 1057–1061. [CrossRef]
38. Hurst, R.; Siyame, E.W.P.; Young, S.D.; Chilimba, A.D.C.; Joy, E.J.M.; Black, C.R.; Ander, E.L.; Watts, M.J.; Chilima, B.; Gondwe, J.; et al. Soil-type influences human selenium status and underlies widespread selenium deficiency risks in Malawi. *Sci. Rep.* **2013**, *3*, 1425. [CrossRef]
39. Joy, E.J.M.; Broadley, M.R.; Young, S.D.; Black, C.R.; Chilimba, A.D.C.; Ander, E.L.; Barlow, T.S.; Watts, M.J. Soil type influences crop mineral composition in Malawi. *Sci. Total Environ.* **2015**, *505*, 587–595. [CrossRef]
40. Manary, M.J.; Abrams, S.A.; Griffin, I.J.; Quimper, M.M.; Shulman, R.J.; Hamzo, M.G.; Chen, Z.; Maleta, K.; Manary, M.J. Perturbed zinc homeostasis in rural 3-5-y-old malawian children is associated with abnormalities in intestinal permeability attributed to tropical enteropathy. *Pediatr. Res.* **2010**, *67*, 671–675. [CrossRef]
41. Bhutta, Z.A. Effect of infections and environmental factors on growth and nutritional status in developing countries. *J. Pediatr. Gastroenterol. Nutr.* **2006**, *43*, S13–S21. [CrossRef]
42. Quihui, L.; Morales, G.G.; Méndez, R.O.; Leyva, J.G.; Esparza, J.; Valencia, M.E. Could giardiasis be a risk factor for low zinc status in schoolchildren from northwestern Mexico? A cross-sectional study with longitudinal follow-up. *BMC Public Health* **2010**, *10*, 85. [CrossRef]
43. de Gier, B.; Mpabanzi, L.; Vereecken, K.; van der Werff, S.D.; D'Haese, P.C.; Fiorentino, M.; Khov, K.; Perignon, M.; Chamnan, C.; Berger, J.; et al. Height, zinc and soil-transmitted helminth infections in schoolchildren: A study in Cuba and Cambodia. *Nutrients* **2015**, *7*, 3000–3010. [CrossRef]
44. Swe, K.M.M.; Abas, A.B.L.; Bhardwaj, A.; Barua, A.; Nair, N.S. Zinc supplements for treating thalassaemia and sickle cell disease. *Cochrane Database Syst. Rev.* **2013**. [CrossRef] [PubMed]
45. Sales, M.C.; de Queiroz, E.O.; Paiva, A.d.A. Association between anemia and subclinical infection in children in Paraíba state, Brazil. *Rev. Bras. Hematol. Hemoter.* **2011**, *33*, 96–99. [CrossRef]
46. Thurnham, D.I.; Mburu, A.S.W.; Mwaniki, D.L.; De Wagt, A. Micronutrients in childhood and the influence of subclinical inflammation. *Proc. Nutr. Soc.* **2005**, *64*, 502–509. [CrossRef] [PubMed]
47. Brown, K.H. Effect of infections on plasma zinc concentration and implications for zinc status assessment in low-income countries. *Am. J. Clin. Nutr.* **1998**, *68*, 425S–429S. [CrossRef] [PubMed]
48. Besecker, B.Y.; Exline, M.C.; Hollyfield, J.; Phillips, G.; DiSilvestro, R.A.; Wewers, M.D.; Knoell, D.L. A comparison of zinc metabolism, inflammation, and disease severity in critically ill infected and noninfected adults early after intensive care unit admission. *Am. J. Clin. Nutr.* **2011**, *93*, 1356–1364. [CrossRef]
49. Thurnham, D.I.; Mccabe, G.P. Influence of infection and inflammation on biomarkers of nutritional status with an emphasis on vitamin A and iron. In *Report: Priorities in the Assessment of Vitamin A and Iron Status in Populations, Panama City, Panama, 15-17 September 2010*; World Health Organization: Geneva, Switzerland, 2012; pp. 63–80.
50. Laillou, A.; Pham, T. Van; Tran, N.T.; Le, H.T.; Wieringa, F.; Rohner, F.; Fortin, S.; Le, M.B.; Tran, D.T.; Moench-Pfanner, R.; et al. Micronutrient deficits are still public health issues among women and young children in Vietnam. *PLoS ONE* **2012**, *7*, e34906. [CrossRef]

51. Tessema, M.; De Groote, H.; Brouwer, I.D.; Feskens, E.J.M.; Belachew, T.; Zerfu, D.; Belay, A.; Demelash, Y.; Gunaratna, N.S. Soil zinc is associated with serum zinc but not with linear growth of children in Ethiopia. *Nutrients* **2019**, *11*, 221. [CrossRef]
52. Beinner, M.A.; Amorim, F.R.D.; Silva, J.B.B.D.; Menezes, M.Â.D.B.C.; Jansen, A.K.; Lamounier, J.A. Plasma zinc and hair zinc levels, anthropometric status and food intake of children in a rural area of Brazil. *Rev. Nutr.* **2011**, *23*, 75–83. [CrossRef]
53. Huey, S.L.; Mehta, S. Stunting: The need for application of advances in technology to understand a complex health problem. *Ebiomedicine* **2016**, *6*, 26–27. [CrossRef]
54. Golden, M.H.N. Malnutrition. In *Textbook of Pediatric Gastroenterology and Nutrition*; Guandalini, S., Ed.; Taylor & Francis: Hampshire, UK, 2004; pp. 424–489. ISBN 9781841843155.
55. Knez, M.; Stangoulis, J.C.R.; Zec, M.; Debeljak-Martacic, J.; Pavlovic, Z.; Gurinovic, M.; Glibetic, M. An initial evaluation of newly proposed biomarker of zinc status in humans - linoleic acid: Dihomo-γ-linolenic acid (LA:DGLA) ratio. *Clin. Nutr. ESPEN* **2016**, *15*, 85–92. [CrossRef]

© 2020 by the authors. Licensee MDPI, Basel, Switzerland. This article is an open access article distributed under the terms and conditions of the Creative Commons Attribution (CC BY) license (http://creativecommons.org/licenses/by/4.0/).
